# Association between anaesthesia-related factors and postoperative neurocognitive disorder: a post-hoc analysis

**DOI:** 10.1186/s12871-023-02318-3

**Published:** 2023-11-10

**Authors:** Manon Stern, Gertrude J. Nieuwenhuijs-Moeke, Anthony Absalom, Barbara van Leeuwen, Hanneke van der Wal-Huisman, Matthijs Plas, Dirk J. Bosch

**Affiliations:** 1 Department of Anesthesiology, University of Groningen, University Medical Center Groningen, Groningen, the Netherlands; 2 Department of Surgery/ Surgical Oncology, University of Groningen, University Medical Center Groningen, Groningen, the Netherlands; 3 Department of Internal Medicine, University of Groningen, University Medical Center Groningen, Groningen, the Netherlands

**Keywords:** Postoperative complications, Postoperative neurocognitive disorder (pNCD), Postoperative cognitive dysfunction (POCD), Anaesthesia

## Abstract

**Background:**

Postoperative neurocognitive disorder (pNCD) is common after surgery. Exposure to anaesthetic drugs has been implicated as a potential cause of pNCD. Although several studies have investigated risk factors for the development of cognitive impairment in the early postoperative phase, risk factors for pNCD at 3 months have been less well studied. The aim of this study was to identify potential anaesthesia-related risk factors for pNCD at 3 months after surgery.

**Methods:**

We analysed data obtained for a prospective observational study in patients aged ≥ 65 years who underwent surgery for excision of a solid tumour. Cognitive function was assessed preoperatively and at 3 months postoperatively using 5 neuropsychological tests. Postoperative NCD was defined as a postoperative decline of ≥ 25% relative to baseline in ≥ 2 tests. The association between anaesthesia-related factors (type of anaesthesia, duration of anaesthesia, agents used for induction and maintenance of anaesthesia and analgesia, the use of additional vasoactive medication, depth of anaesthesia [bispectral index] and mean arterial pressure) and pNCD was analysed using logistic regression analyses. Furthermore, the relation between anaesthesia-related factors and change in cognitive test scores expressed as a continuous variable was analysed using a z-score.

**Results:**

Of the 196 included patients, 23 (12%) fulfilled the criteria for pNCD at 3 months postoperatively. A low preoperative score on Mini-Mental State Examination (OR, 8.9 [95% CI, (2.8–27.9)], *p < 0.001*) and a longer duration of anaesthesia (OR, 1.003 [95% CI, (1.001–1.005)], *p = 0.013*) were identified as risk factors for pNCD. On average, patients scored higher on postoperative tests (mean z-score 2.35[± 3.13]).

**Conclusion:**

In this cohort, duration of anaesthesia, which is probably an expression of the complexity of the surgery, was the only anaesthesia-related predictor of pNCD. On average, patients’ scores on cognitive tests improved postoperatively.

## Introduction

Postoperative neurocognitive disorder (pNCD) is often a subtle impairment of memory, concentration, and information processing, persisting for weeks or months after surgery [1]. It commonly occurs after surgery, with an incidence between 10 and 16% at 3 months postoperatively in patients aged ≥ 55 who undergo noncardiac surgery [2–4]. Postoperative NCD is especially prevalent among elderly patients and, with an increasing number of multimorbid older adults undergoing surgery, pNCD is expected to present an increasing issue for public health. The consequences of pNCD are not only an association with increased risk of mortality and prolonged hospitalisation, but also disability, reduced self-sufficiency, and decreased quality of life [2, 5].

Much research has been dedicated to the identification of risk factors for the development of pNCD. Increasing age, a lower educational level and pre-existing cognitive disorders are well known risk factors for pNCD [2, 6, 7]. The relationship between pNCD and anaesthesia-related factors is, however, less clear. A recent meta-analysis indicated that propofol-based total intravenous anaesthesia (TIVA) is associated with better cognitive outcomes in the first postoperative month compared with inhalational anaesthesia, which might be explained by the positive effects of propofol in attenuating the (neuro)inflammatory response [8, 9]. It is unknown whether this is also the case for NCD at 3 months postoperatively [8]. Moreover, the impact of intraoperative depth of anaesthesia on postoperative cognitive function continues to be controversial, with some studies indicating a protective effect of high bispectral index (BIS) against NCD at 3 months postoperatively [10–12]. In the BALANCED delirium study, for instance, a BIS target of 50 resulted in a significant decrease in the incidence of postoperative delirium (POD) when compared with a BIS target of 35, and the former was associated with a lower incidence of NCD at 1 year postoperatively [12]. Intraoperative hypotension was not related to pNCD in a meta-analysis of 700 patients [13]. In contrast, duration of anaesthesia and surgery is a factor that is consistently associated with pNCD [3]. The overall association between long-term cognitive function and anaesthesia-related factors, such as type of anaesthesia, as well as intraoperative parameters (e.g., blood pressure, depth of anaesthesia) remains uncertain.

Therefore, the aim of this study was to identify potential anaesthesia-related risk factors for NCD at 3 months after surgery.

## Materials and methods

### Protocol

This study is a post-hoc analysis of the PICNIC study (PostoperatIve Cognitive dysfunctioN In elderly Cancer patients [PICNIC], trial number NL31486.042.10), a prospective observational study conducted at the University Medical Center Groningen (UMCG, Groningen, NL) [14–19]. The PICNIC study was approved by the Medical Ethical Committee of the UMCG and data collection was conducted in accordance with the Declaration of Helsinki. All subjects provided written informed consent.

### Patients and clinical data collection in the PICNIC study

**Fig. 1:**
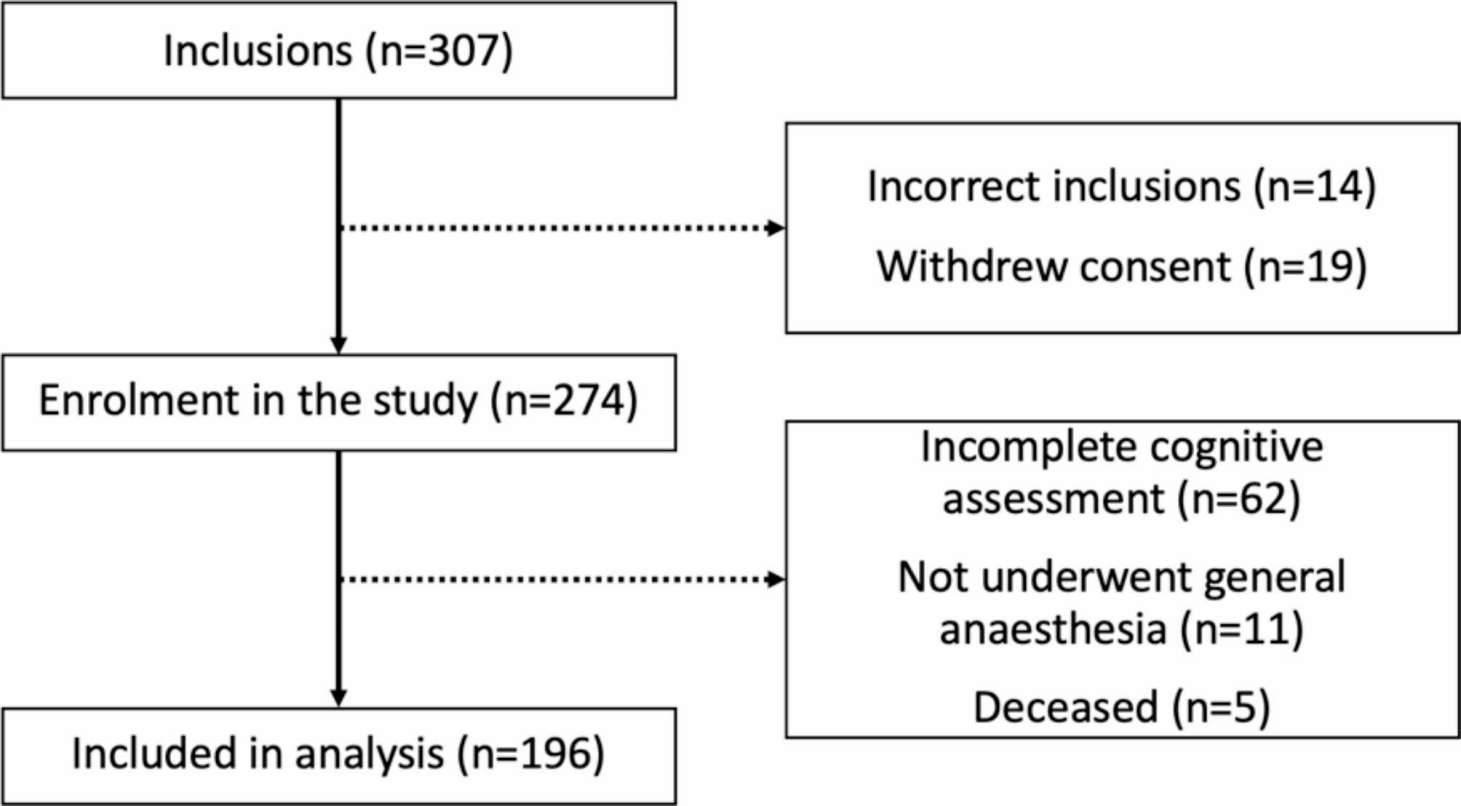
Consort Diagram. Of the 307 included patients in the PICNIC study, 33 were excluded before surgery. Three months postoperatively, 62 patients were excluded due to incomplete cognitive assessment. Five patients died, and 11 patients were excluded in the current analysis because they did not undergo general anaesthesia. The sample of the current analysis included 196 patients

The cohort of the PICNIC study consisted of patients aged ≥ 65 years, scheduled for surgery for a solid malignant tumour at the UMCG between 2010 and 2014 (see Fig. 1). Patients were excluded from the PICNIC study if they had a physical condition potentially impeding compliance with the study procedures, for instance severe visual or auditory impairment, recent stroke or preoperative cognitive deficits. Furthermore, patients were excluded if their understanding of the Dutch language was insufficient.

Prospectively, the following data were collected: age, sex, BMI, tumour type, disease stage, and surgical characteristics. The Groningen Frailty Index (GFI) was used preoperatively to assess patients’ condition with regards to the physical, cognitive, social and psychological domains [20]. Furthermore, Charlson Comorbidity score (CCS) and Mini-Mental State Examination (MMSE) were used preoperatively to classify comorbidities and measure cognitive impairment, respectively [21, 22]. Anaesthesia and procedure related relevant intraoperative data were recorded perioperatively. Details of complications occurring up to 30 days postoperatively were recorded and classified according to the Clavien-Dindo Classification (POD excluded).

### Anaesthesia-related factors

To analyse the association between anaesthesia and cognitive function at 3 months postoperatively, several factors were taken into account. First, the association between type of anaesthesia (general versus combined general and neuraxial anaesthesia) and pNCD was analysed. Second, the association between depth of anaesthesia quantified as the bispectral index (BIS) and pNCD was assessed. We analysed the effect of mean BIS between induction and end of surgery, the total time BIS < 45, the percentage time BIS < 45, the total time BIS > 60 as well as the percentage time BIS > 60. A BIS < 45 was chosen as a cut-off as it is a commonly used definition for deep anaesthesia and has been shown to increase the risk of adverse postoperative outcomes [23, 24]. A BIS > 60 was chosen as an upper limit, because it is usually recommended to avoid values higher than 60 [25]. Furthermore, the risk of having pNCD was compared between patients with a mean intraoperative BIS < 45 and patients with a mean intraoperative BIS > 45. Additionally, the effects of different sedatives and analgesics used during induction and maintenance of anaesthesia were examined. Other factors that were taken into account included the duration of anaesthesia, the total time mean arterial pressure (MAP) < 70, the area under the curve (AUC) MAP < 70, and if the patient received additional vasoactive medication during anaesthesia. Blood pressure and BIS were only included in the analysis if data was available for at least 85% of the total duration of surgery.

### Cognitive tests

Cognitive function was evaluated at baseline (2 weeks before surgery) and at 3 months postoperatively using different neuropsychological tests. Three cognitive domains were assessed: memory, executive functioning and information processing speed. Memory function was assessed using the Dutch versions of the Rey’s Auditory Verbal Learning Test (RAVLT) immediate recall and the RAVLT delayed recall [26]. The outcome of the RAVLT was defined by the total number of words (maximum of 75) correctly remembered during the five immediate recall trials (lowest score, 0; highest score, 75) and the total number of words (maximum of 15) remembered at the delayed recall trial (lowest score, 0; highest score, 15). For assessment of executive function, the Ruff’s Figural Fluency Test (RFFT) and the Trail Making Test part B (TMT-B) were used [27, 28]. Performance on the RFFT was expressed by the total number of unique designs drawn in parts 1 to 5 (lowest score: 0; highest score: 175). For the TMT-B, the number of seconds it took to complete the test was used as the outcome variable (lowest score: 0; highest score: 480). Performance with regards to information processing speed was determined using the Trail Making Test part A (TMT-A) [27]. The number of seconds it took to complete the TMT-A (lowest score, 0; highest score, 480) was used as the outcome variable.

### Definition of postoperative NCD

Postoperative NCD was defined as a decline ≥ 25%, relative to the baseline score, in the scores of ≥ 2 of the aforementioned cognitive tests at 3 months postoperatively. In addition, change in score on postoperative cognitive tests as compared to preoperative tests was expressed as a continuous variable by means of z-scores, with a positive z-score indicating an overall increased outcome on cognitive tests and a negative score indicating a decline. This method takes into account a general deterioration in all tests as well as a substantial deterioration in only one or some tests and enables the analysis of the overall change in scores on the postoperative cognitive tests as compared to the preoperative tests.

### Data analysis and statistics

As the present study is a secondary analysis of a pre-existing database, no power calculation was conducted for this analysis and the sample size was based on available data. Data with regards to patient characteristics and anaesthesia-related factors are presented as frequencies with percentages for categorical and ordinal variables. Median and interquartile range (IQR) are used for continuous, not normally distributed data and mean and standard deviation (SD) are used for continuous, normally distributed data. Univariate and multivariate logistic regression analysis were performed to analyse the association between patient characteristics and pNCD as well as between anaesthesia-related factors and pNCD. Odds ratios (ORs) and their corresponding 95% confidence intervals (95% Cis) are reported. Variables with P values of < 0.15 on univariate analyses were included in the multivariate analysis. Hosmer-Lemeshow goodness-of-fit test was used to evaluate model fit. Furthermore, change in performance on each cognitive test was assessed as a continuous variable by using a z-score. For this purpose, a change score (postoperative minus baseline score) was calculated for each separate test and each patient. For the Trail Making Test the sign was corrected, so that a higher z-score indicated cognitive improvement. The z-score for each patient and each test was subsequently calculated by dividing the change score by the standard deviation of the group’s mean change score for the corresponding test. Lastly, per patient, a combined z-score was derived by adding the z-scores of the individual tests, enabling cognitive function to be expressed as a continuous variable with a positive value indicating a postoperative improvement in cognitive tests. The association between anaesthesia-related factors and change in cognitive function as expressed by the z-scores was analysed in a univariate analysis using Spearman’s rank correlation coefficient for continuous explanatory variables and using independent samples t test and ANOVA tests for nominal explanatory variables with 2 or > 2 categories respectively. A p-value of < 0.05 was considered statistically significant. All analyses were performed using the Statistical Package for Social Sciences: SPSS version 20.0.0 (SPSS Inc., Chicago, DL USA).

## Results

Of the 307 included patients, a total of 196 patients fulfilled all inclusion criteria (Fig. 1*)*. Preoperatively, 14 patients were included incorrectly, for instance due to cancellation of surgery, and 19 withdrew consent retrospectively. Postoperatively, 62 patients were excluded due to incomplete cognitive assessment (mainly for logistical reasons), 11 did not undergo general anaesthesia and 5 deceased during the research period (Fig. 1).

The mean age of included patients was 72 (± 6) and 50.5% were female. The majority of patients had a preoperative GFI score ≤ 3 (75.7%) and a score > 26 on MMSE (91.3%). Of all patients, 70.8% had a CCS > 2 and 57.2% presented with a disease stage < 3. In total, 23 (12%) of the 196 patients met the criteria for pNCD at 3 months postoperatively. A summary of patient characteristics and anaesthesia-related factors is shown in Table 1.

**Table 1:** Patient characteristics and anaesthesia-related factors

Patient characteristics	Overall n = 196	Postoperative NCD n = 23 (11.7%)	No Postoperative NCD n = 173 (88.3%)	P
Age	72 ± 6	75 ± 7	72 ± 6	0.145
Gender				
-Female -Male	99 (50.5) 97 (49.5)	11 (47.8) 12 (52.2)	88 (50.9) 85 (49.1)	0.784
GFI				
-<= 3 ->3	128 (75.7) 41 (24.3)	14 (70.0) 6 (30.0)	114 (76.5) 35 (23.5)	0.580
MMSE				
-<= 26 ->26	17 (8.7) 179 (91.3)	7 (30.4) 16 (69.6)	10 (5.8) 163 (94.2)	0.001
CCS				
-<= 2 ->2	57 (29.2) 138 (70.8)	6 (27.3) 16 (72.7)	51 (29.5) 122 (70.5)	0.830
Disease stage				
-Benign, I, II -III, IV	111 (57.2) 83 (42.8)	18 (78.3) 5 (21.7)	93 (54.4) 78 (45.6)	0.030
Highest postoperative complication according to Clavien-Dindo (without delirium)				
-No complication -Grade I -Grade II -Grade III -Grade IV	103 (52.6) 17 (8.7) 50 (25.5) 18 (9.2) 8 (4.1)	10 (43.5) 1 (4.3) 6 (26.1) 2 (8.7) 4 (17.4)	93 (53.8) 16 (9.2) 44 (25.4) 16 (9.2) 4 (2.3)	0.015
**Anaesthesia-related factors**				
Type anaesthesia				
-General -Combined general and neuraxial anaesthesia	93 (47.4) 103 (52.6)	10 (43.5) 13 (56.5)	83 (48.0) 90 (52.0)	0.685
Duration of anaesthesia	203土217	280土260	195土218	0.023
Agents induction anaesthesia				
-Propofol -Other	181 (94.3) 11 (5.7)	19 (90.5) 2 (9.5)	162 (94.7) 9 (5.3)	0.344
Agents induction analgesia				
-Sufentanil -Remifentanil -other	166 (86.5) 21 (10.9) 5 (2.6)	20 (95.2) 1 (4.8) 0 (0)	146 (85.4) 20 (11.7) 5 (2.9)	0.439
Agents maintenance anaesthesia				
-Propofol -Isoflurane -Sevoflurane -Other	130 (68.4) 13 (6.8) 35 (18.4) 12 (6.3)	17 (77.3) 2 (9.1) 3 (13.6) 0 (0)	113 (67.3) 11 (6.5) 32 (19.0) 12 (7.1)	0.503
Agents maintenance analgesia				
-Sufentanil -Remifentanil -Other	144 (75.0) 27 (14.1) 21 (10.9)	17 (77.3) 4 (18.2) 1 (4.5)	127 (74.7) 23 (13.5) 20 (11.8)	0.537
Additional vasoactive medication received				
-No -Yes	11(5.6) 184 (94.4)	1 (4.3) 22 (95.7)	10 (5.8) 162 (94.2)	1.000
Mean BIS	43土11	42土11	43土12	0.786
Mean BIS				
-<45 ->=45	53 (60.2) 35 (39.8)	10 (66.7) 5 (33.3)	43 (58.9) 30 (41.2)	0.549
BIS < 45 time	119土141	127土179	115土145	0.504
BIS < 45%	63土55	62土63	62土53	1.000
BIS > 60 time	7土14	6土14	8土14	1.000
BIS > 60%	4土8	3土12	4土8	0.584
MAP < 70 time	47土68	68土73	45土65	0.386
MAP 70 AUC	433土644	606土655	419土640	0.773

*Notes.* Categorical and ordinal variables are expressed as absolute frequencies with percentages (%); continuous, not normally distributed data are expressed as median plus IQR; continuous, normally distributed data are expressed as mean plus SD

The results of the logistic regression analyses for pNCD are shown in Table 2. After adjusting for other variables, a preoperative score ≤ 26 on MMSE (OR, 8.9 [95% CI, (2.8–27.9)], *p < 0.001*) and longer duration of anaesthesia (OR, 1.003 [95% CI, (1.001–1.005)], *p = 0.013*) were identified as risk factors of pNCD. The results of the Hosmer-Lemeshow goodness-of-fit test were not significant *(*χ^2^ = 5.122 *p = 0.744)*, indicating that the model provides a good fit to the data.

**Table 2:** Patient characteristics and anaesthesia-related factors related to postoperative NCD (outcomes of the univariate and multivariate regression analyses)

Patient characteristics	Univariate OR (95%CI)	P	Multivariate OR (95%CI)	P
Age	1.0 (1.0–1.1)	0.078*		
Gender				
-Female -Male	1 1.1 (0.5–2.7)	0.784		
GFI				
-<= 3 ->3	1 1.4 (0.5–3.9)	0.525		
MMSE				
->26 -<= 26	1 7.1 (2.4–21.3)	**< 0.001**	1 8.9 (2.8–27.9)	**< 0.001**
CCS				
-<= 2 ->2	1 1.1 (0.4-3.0)	0.046*		
Highest Postoperative complication (ClavienDindo without delirium and death)				
-No complication -Grade I -Grade II -Grade III -Grade IV	1 0.6 (0.1–4.9) 1.3 (0.4–3.7) 1.2 (0.2–5.8) 9.3 (2.0–43.0)	0.061*		
**Anaesthesia-related factors**				
Type anesthesia				
-General -Combined general and neuraxial anaesthesia	1 1.2 (0.5–2.9)	0.685		
Duration of anaesthesia	1.003 (1.000-1.005)	**0.027**	1.003 (1.001–1.005)	**0.013**
Agents induction anaesthesia				
-Propofol -Other	1 1.4 (0.6–3.1)	0.435		
Agents induction analgesia				
-Sufentanil -Remifentanil -other	1 0.4 (0-2.9) 0 (0)	0.632		
Agents maintenance anaesthesia				
-Propofol -Isoflurane -Sevoflurane -Other	1 1.2 (0.2–5.9) 0.6 (0.2–2.3) 0 (0)	0.891		
Agents maintenance analgesia				
-Sufentanil -Remifentanil -Other	1 1.3 (0.4–4.2) 0.4 (0–3.0)	0.561		
Additional vasoactive medication received				
-No -Yes	1 1.4 (0.2–11.1)	0.776		
Mean BIS	1.0 (0.9–1.1)	0.691		
Mean BIS				
-<45 ->=45	1 0.7 (0.2–2.3)	0.551		
BIS < 45 time	1.0 (1.0–1.0)	0.380		
BIS < 45%	1.0 (1.0–1.0)	0.803		
BIS > 60 time	1.0 (1.0–1.0)	0.729		
BIS > 60%	1.0 (0.9-1.0)	0.536		
MAP < 70 time	1.0 (1.0–1.0)	0.716		
MAP 70 AUC	1.0 (1.0–1.0)	0.886		

*Notes.* Factors with a p value < 0.15 on univariate analyses were included in the multivariate analyses. ∗nonsignificant in multivariate analyses

**Fig. 2:**
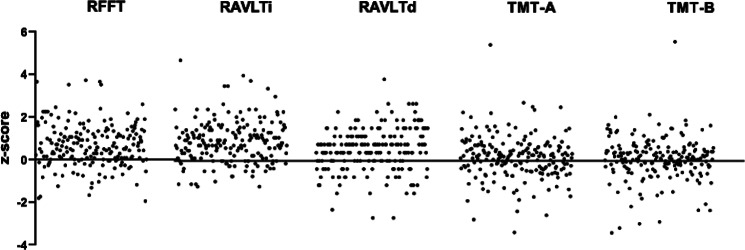
Distribution of z-scores for each cognitive test

Outcomes of the analysis of the association between anaesthesia-related factors and change in scores on cognitive tests as expressed by a z-score are displayed in Table 3. No statistically significant relation between any of the anaesthesia-related factors and change in score on cognitive tests was seen. The mean z-score of all patients was 2.35 (± 3.13), indicating that, on average, patients scored higher on postoperative tests as compared to preoperative tests. The distribution of z-scores among the different neuropsychological tests is shown in Fig. 2.

**Table 3:** Association between anaesthesia-related factors and change in cognitive function as a continuous outcome

Variable	Mean z-score	Test statistic	P
Type anaesthesia			
-General -Combined general and neuraxial anaesthesia	2.38土2.89 2.32土3.34	T = 0.124	0.901
Duration of anaesthesia		R = -0.075	0.299
Agents induction anaesthesia			
-Propofol -Other	2.35土3.13 2.82土2.80	T = -0.488	0.626
Agents induction analgesia			
-Sufentanil -Remifentanil -Other	2.40土3.19 2.63土2.48 0.70土2.41	F = 0.799	0.451
Agents maintenance anaesthesia			
-Propofol -Isoflurane -Sevoflurane -Other	2.15土2.68 2.17土4.44 2.57土4.13 4.14土2.35	F = 1.577	0.197
Agents maintenance analgesia			
-Sufentanil -Remifentanil -Other	2.55土3.17 1.42土3.14 2.34土2.65	F = 1.494	0.227
Additional vasoactive medication received			
-Yes -No	2.28土3.18 3.17土1.94	T = 0.912	0.363
Mean BIS		R = 0.059	0.586
Mean BIS			
->=45 -<45	1.77土3.71 1.82土3.45	T = 0.062	0.951
BIS lower 45 time		R = − 0.122	0.261
BIS lower 45%		R = -0.100	0.355
BIS higher 60 time		R = 0.036	0.743
BIS higher 60%		R = 0.092	0.399
MAP lower 70 time		R = -0.109	0.286
MAP 70 AUC		R = -0.078	0.443

*Notes.* Z-scores are expressed as mean with standard deviation; p-values with respect to Spearman’s rank correlation coefficient, independent samples t-test and ANOVA.

## Discussion

The aim of this study was to identify potential anaesthesia-related risk factors for NCD at 3 months postoperatively. In this cohort, duration of anaesthesia was the only anaesthesia-related factor that was significantly associated with pNCD at 3 months. Our study confirmed the known association between pNCD and poor baseline cognitive function. On average, patients’ postoperative cognitive performance scores improved compared to preoperative scores.

Exposure to anaesthetic agents has been implicated as a potential cause of pNCD [6]. Although a recent meta-analysis indicated that the use of propofol-based TIVA is associated with better cognitive outcomes at 30 days postoperatively as compared to inhalational anaesthetics, no beneficial effect of propofol was found at 3 months postoperatively [8, 29, 30]. Also in our cohort, choice of anaesthetic drugs did not affect the risk of pNCD, suggesting that the type of anaesthetic may, at least be related to the short-term cognitive function. Moreover, pNCD has also been detected in patients who received spinal anaesthesia without sedation, indicating that the aetiology of pNCD is likely to be multifactorial [6, 31].

While the impact of anaesthetic agents on the risk of developing pNCD remains uncertain, duration of surgery and consequently anaesthesia is likely to influence the risk of pNCD [7]. This was also shown in our cohort in which a longer duration of anaesthesia was associated with a higher risk of pNCD. However, as the duration of anaesthesia may be an indicator of the complexity and invasiveness of a surgery, it is likely that not the duration itself, but instead the extent of the intervention and the potentially associated systemic inflammation increases the risk of pNCD.

In addition to the type of anaesthetic agent and duration of anaesthesia, depth of anaesthesia did not affect the risk of pNCD in our cohort. While some studies have shown that depth of anaesthesia monitoring reduces the incidence of POD, possibly by reducing the time of deep anaesthesia, the effect of deep anaesthesia on pNCD is still inconclusive [32–34]. In contrast to our findings, however, a meta-analysis by *Ling et al.* demonstrated a protective effect of higher BIS values for pNCD at 3 months postoperatively [10]. This difference may be explained by differences in the cut-offs used. While we compared the risk of pNCD between patients with a mean BIS < 45 and > 45, BIS targets in studies included in the meta-analysis ranged from 30 to 45 to 40–50 for patients randomised to the deep anaesthesia groups and from 46 to 55 to 55–65 for patients in the light anaesthesia groups. Furthermore, the meta-analysis included studies investigating differences in the incidence of pNCD between BIS-guided and non-BIS-guided anaesthesia instead of between different anaesthesia depths. This may have influenced the analysis, as patients in the non-BIS-guided group were likely to be exposed to longer periods of very deep anaesthesia.

The association between pNCD and different opioids used during surgery has also been studied. Previously it was hypothesised that short-acting opioids decrease the risk of postoperative delirium compared to long-acting opioids, potentially by decreasing length of ICU and hospital stay [35–37]. However, in a study comparing the incidence of cognitive impairment at postoperative day 1, 4 and 30 in patients who underwent elective coronary artery bypass grafting, no difference was found between remifentanil- and sufentanil-based anaesthesia [37]. Likewise, we did not demonstrate an association between pNCD and the agents used for induction and maintenance of analgesia.

Although there does not seem to be a relationship between pNCD and the type of opioid used, an increased opioid dose may be related to a higher risk of postoperative cognitive impairment [38]. Similarly, previous studies implied that, possibly by limiting the amount of opioids used, regional anaesthesia decreases the incidence of postoperative cognitive impairment in the first postoperative week as compared to general anaesthesia [39]. In contrast, a more recent study could not demonstrate a protective effect of regional anaesthesia on postoperative delirium and postoperative cognitive impairment in the first postoperative week [40]. In our study, the comparison of combined general and neuraxial anaesthesia with general anaesthesia alone did not affect the risk of pNCD.

For a long time, pNCD was believed to be a direct consequence of intraoperative physiological perturbations, such as hypoxaemia or hypotension [6]. After publication of the ISPOCD study, however, this was considered unlikely, as neither hypoxaemia nor hypotension were shown to be associated with the risk of developing pNCD [3]. Similar results were found in a meta-analysis, in which intraoperative hypotension was not associated with pNCD [13]. Accordingly, we did not see a relationship between pNCD and the time MAP < 70 and MAP 70. Comparison is nonetheless difficult, due to the great heterogeneity of definitions used for intraoperative hypotension [41]. Moreover, the use of vasoactive medication during surgery did not affect the risk of pNCD.

Most studies of postoperative cognitive function have used a binary outcome – pNCD present or not. This leads to a focus on the patients who do develop NCD, whereas, as shown in our study, many patients have better cognitive function after surgery. While the higher postoperative scores may be interpreted as a learning effect (although there were more than 3 months between the 5 different cognitive tests), it is likely that patient’s cognitive function was impaired preoperatively. Possible causes for worse cognitive function before surgery are anxiety and stress related to the upcoming surgery or due to previous chemotherapy. On the other hand, once surgery has been successfully concluded, and patients have recovered from their surgery, improved physical and mental wellbeing might improve their performance on cognitive function tests [42–44].

In our cohort, 12% of the patients fulfilled the criteria for NCD at 3 months postoperatively. Although it is difficult to compare the incidence of pNCD between studies, due to, for instance, differences in the definition of pNCD and differences in type and number of cognitive tests administered, the incidence seen in our study seems to be in line with previous findings. In previous studies of patients undergoing noncardiac surgery, incidences of 10% (2 SD method, patients aged ≥ 60), 13% (2 SD method, patients aged ≥ 60) and 16% (reliable change method, patients aged ≥ 55) have been documented [2–4]. In our study, 22% of the enrolled patients did not undergo cognitive assessment at 3 months follow-up, due to a combination of reasons, including logistic reasons and self-reported overall health status. Overall, we expect to provide a very accurate representation of the incidence of pNCD.

While many studies have investigated the effects of anaesthesia on the development of cognitive dysfunction in the early postoperative phase, we focused on the effects on cognitive function at 3 months postoperatively, as these are probably associated with a greater impact on a patient’s life. However, there are a few relevant limitations to the applicability of our findings. As the present study is a secondary analysis of a pre-existing database, no sample size calculation was performed, and it was not designed to find a relationship between anaesthesia-related factors and pNCD. Furthermore, our data was collected several years ago. While we do not think that this affects the applicability of our findings, as at that time BIS monitoring was standard of care and the majority of patients received TIVA with propofol, our definition of pNCD differs from the more recently published recommendations for the nomenclature of cognitive change associated with anaesthesia and surgery [45]. This impairs the comparability of our findings to more recent studies. Generally, studies investigating pNCD differ greatly with regards to diagnostic rules, cut-offs, timing of follow-up and differences in the tests used, with a recent meta-analysis reporting no less than 259 different cognitive tests in 274 studies [46]. Although our chosen definition of pNCD (a decline of ≥ 25% relative to baseline in ≥ 2 tests) differs from the latest recommendations, we believe that our method is a very accurate representation of the incidence of pNCD. In the current study, extensive cognitive testing was performed 3 months postoperatively to avoid interference with surgery/anaesthesia related factors like medication or fatigue. The definition of pNCD as a change in two or more cognitive tests as compared to baseline is commonly used in studies reporting NCD as a dichotomous outcome [46]. An advantage of the criteria we used for diagnosing pNCD is that we performed multiple different cognitive tests and thereby assessed functioning in several cognitive domains. Lastly, as we did not use a control group, the impact of a learning effect, normal age-related cognitive decline and other possible unknown confounders were not reflected in the analysis.

In conclusion, duration of anaesthesia appeared to be the only anaesthesia-related factor significantly associated with pNCD at 3 months. This outcome, however, is more likely to indicate that the extent of surgery might be related to the risk of developing pNCD rather than duration anaesthesia being the cause of pNCD. In general, when applying balanced anaesthesia, there do not seem to be any anaesthesia-related factors that influence the risk of pNCD. In fact, on average patients’ postoperative cognitive performance scores were improved compared to preoperative scores.

## Data Availability

The datasets used and/or analyzed during the current study are available from the corresponding author on reasonable request.

## Abbreviations

AUC: Area under the curve
BIS: Bispectral index
CCS: Charlson Comorbidity Score
CI: Confidence interval
GFI: Groningen Frailty Index
IQR: Interquartile range
MAP: Mean arterial pressure
MMSE: Mini-Mental State Examination
NCD: Neurocognitive disorder
OR: Odds ratio
POD: Postoperative delirium
RAVLT: Rey’s Auditory Verbal Learning Test
RFFT: Ruff’s Figural Fluency Test
TIVA: total intravenous anaesthesia
TMT-A: Trail Making Test part A
TMT-B: Trail Making Test part B
